# Effect of Cup Material on *S*-Methoprene Bioavailability and Susceptibility Estimates in Larval Mosquito Bioassays

**DOI:** 10.3390/pathogens15070769

**Published:** 2026-07-22

**Authors:** Sarah S. Wheeler, Kara Kelley, Mario Novelo

**Affiliations:** Sacramento-Yolo Mosquito and Vector Control District, 8631 Bond Rd, Elk Grove, CA 95624, USA

**Keywords:** *S*-methoprene, larval resistance monitoring, expanded polystyrene, assay standardization, vector management, *Culex quinquefasciatus*

## Abstract

Mosquito control programs rely on larvicides such as S-methoprene to suppress vector populations and prevent mosquito-borne diseases including West Nile virus (WNV). Resistance surveillance is essential for maintaining the long-term effectiveness of larvicide active ingredients and is typically conducted using cup bioassays to evaluate larval susceptibility. Disposable Styrofoam cups are commonly used for these assays because they minimize adsorption of S-methoprene under standard testing conditions. However, recent restrictions on expanded polystyrene products in California necessitated the identification and evaluation of suitable replacement cup materials for larval resistance bioassays. Four alternative cup materials, high-density polyethylene (HDPE), polyethylene terephthalate (PET), polypropylene (PP), and paper cups, were compared to Styrofoam cups. Cup bioassays were conducted using a laboratory colony of *Culex quinquefasciatus* exposed to *S*-methoprene concentrations of 1–40 ppb. Emergence inhibition data were analyzed using probit regression to estimate the concentrations causing 50% and 90% emergence inhibition (LC_50_ and LC_90_, respectively) for each cup type. Cup material significantly affected concentration-response relationships. HDPE and paper cups produced significantly higher LC_50_ and LC_90_ estimates than Styrofoam, whereas PET and PP produced responses more comparable to the Styrofoam reference. Concentration-response slopes were similar among cup materials, indicating that cup material primarily shifted the concentration required to achieve a given level of emergence inhibition. These findings demonstrate that cup material can significantly influence estimated S-methoprene LC values. Replacement assay cups should therefore be validated before implementation in larval resistance monitoring programs.

## 1. Introduction

Mosquito-borne diseases are a major public health concern worldwide. To mitigate the threat of insecticide resistance within mosquito populations, mosquito control agencies implement integrated mosquito management (IMM) programs [1,2,3] that utilize a multipronged approach including public education, immature mosquito habitat source reduction, biological control, microbial control, and, when necessary, chemical control. To reduce selection pressure for resistance, larvicides and adulticides are applied in a targeted manner, and insecticide classes are rotated when possible. Routine insecticide resistance monitoring is also an essential component of IMM because it provides the information needed to detect changes in susceptibility and guide resistance management [4].

Larvicides are an important component of IMM programs and often serve as the primary method of mosquito suppression because they target immature mosquito life stages in aquatic larval habitats and reduce the need for wide-area adult mosquito control insecticide applications. Mosquito larvicides include microbial products, insect growth regulators (IGRs), and surface films, each with distinct modes of action and operational considerations. Among these products, S-methoprene, a juvenile hormone analog (JHA), is widely used because of its low toxicity to non-target organisms [5] and effectiveness against immature mosquitoes. Juvenile hormone analogs interfere with normal mosquito larval development by mimicking juvenile hormones present during aquatic stages [6,7], preventing the emergence of adult mosquitoes [8].

Larval resistance can be assessed using concentration-response bioassays commonly referred to as cup bioassays [9,10,11]. Cup bioassays are a standard tool for estimating mosquito susceptibility to larvicides and are typically conducted using reusable glass bowls and beakers or disposable cups to minimize contamination [11]. Because standardized bioassays are intended to provide reproducible estimates of larval susceptibility, factors that alter the effective larvicide concentration may introduce unintended variation into assay results. *S*-methoprene readily adsorbs to certain substrates [12,13], potentially reducing its concentration in solution and influencing measured concentration-response relationships. Consequently, early bioassays used silanized glassware to minimize losses of active ingredient [14]. Subsequent procedures transitioned to disposable Styrofoam cups [9,10,15], because they are inexpensive, disposable, and appear to minimize reductions in *S*-methoprene bioavailability under standard assay conditions.

Recently, California Senate Bill 54 (SB 54; Solid Waste: Reporting, Packaging, and Plastic Food Ware; 2022) restricted the sale of expanded polystyrene (Styrofoam) food-service products, resulting in the unavailability of Styrofoam cups in California. Alternative cup materials therefore required evaluation before they could be adopted for standardized *S*-methoprene larval bioassays. Accordingly, four candidate replacement cup materials were compared with Styrofoam to determine whether cup material influenced measured *S*-methoprene concentration-response relationships and to identify a suitable replacement for standardized larval bioassays.

## 2. Materials and Methods

### 2.1. Mosquitoes

Mosquito larvae were reared under laboratory-controlled conditions at 25 °C to ensure uniform larval development. Experiments were conducted using a laboratory reference colony of *Culex quinquefasciatus* (CQ1) originally collected in Merced County, CA, in the 1950s [16]. Mosquitoes were reared in plastic trays (24 cm × 30 cm) containing 1.5 L of dechlorinated tap water. Larvae were fed 0.2 g of finely ground TetraMin^®^ (47.6% by volume; Tetra, Blacksburg, VA, USA) per tray daily. Prior to each bioassay, larvae from all rearing trays were combined to produce a uniform age-matched cohort, and late third- to early fourth-instar larvae were selected for testing.

### 2.2. Larvicide Bioassays

Five cup materials were evaluated: Styrofoam (237 mL, Dart, Mason, MI, USA) high-density polyethylene (HDPE) (237 mL, Mercedes Scientific, Lakewood Ranch, FL, USA), polyethylene terephthalate (PET) (355 mL, First Street available at Smart and Final, Los Angeles, CA, USA), polypropylene (PP) (251 mL, Karat by Lollicup, Chino, CA, USA) and Flexstyle^®^ paper food containers lined with polyethylene (473 mL, Dart, Mason, MI, USA).

Bioassays were conducted based on previously published methods [9,10,15] with some modifications. For each bioassay cup, 25 larvae were placed in a 90 mL condiment cup containing 10 mL of distilled water. Each test cup was filled with 90 mL of distilled water. Five percent Altosid^®^ Liquid Larvicide (Wellmark International, Schaumburg, IL, USA) was serially diluted immediately before each assay to produce final S-methoprene concentrations of 0, 1, 2, 5, 10, 20, and 40 ppb. Four technical replicates were prepared for each cup material and concentration combination.

After S-methoprene was added, the larvae and 10 mL of water from the condiment cup were transferred to each test cup, producing a final assay volume of 100 mL. Each cup received 37 mg of coarsely ground TetraMin. Cups were fitted with a clear lid, and all openings were closed with window screening. The assay cups were held in a climate-controlled rearing space (24 °C).

Assays were first examined beginning 5 days after treatment and were scored after all pupae had either successfully emerged or died, typically 7 days after treatment. Successful adult emergence was determined by the presence of an exuvium. Pupae that died before emergence and adults that failed to completely emerge were classified as emergence inhibited. Living larvae remaining at the end of the assay were excluded from the analysis because they had not completed development during the observation period. Individuals that successfully completed adult emergence were classified as not emergence inhibited. Emergence inhibition was used as the response variable in all analyses. The complete experiment was conducted on five separate occasions.

### 2.3. Statistical Analysis

Statistical analyses were conducted in R version 4.6.0 [17]. Control emergence inhibition was evaluated prior to analysis and remained below thresholds requiring Abbott’s correction. Water-only controls were excluded from subsequent concentration-response analyses.

To estimate the S-methoprene concentrations associated with 50% and 90% emergence inhibition for each cup material, probit models were fit using the ecotox package (v1.4.4) [18]. The models characterized the relationship between *S*-methoprene concentration and emergence inhibition and were used to estimate LC_50_ and LC_90_ values with 95% confidence intervals. Relative LC ratios were calculated by dividing the LC_50_ and LC_90_ estimates for each cup material by the corresponding estimates obtained using Styrofoam cups.

To evaluate the effect of cup material on concentration-response relationships, emergence inhibition was analyzed using a generalized linear model with a quasibinomial error distribution and probit link. The model included log-transformed S-methoprene concentration, cup material, and experimental replicate as fixed effects. The contribution of cup material to emergence inhibition was evaluated by comparing models with and without the cup material term using an analysis-of-deviance F-test.

To determine whether the effect of cup material changed across the range of S-methoprene concentrations, a second generalized linear model including a cup material × concentration interaction was compared with the main-effects model (concentration, cup material, and experimental replicate) using an analysis-of-deviance F-test. This analysis evaluated whether cup material affected the slope of the concentration-response relationship in addition to shifting the concentration required to achieve a given level of emergence inhibition. Cup-specific concentration-response slopes were also estimated using separate probit models for descriptive purposes.

**Figure 1:**
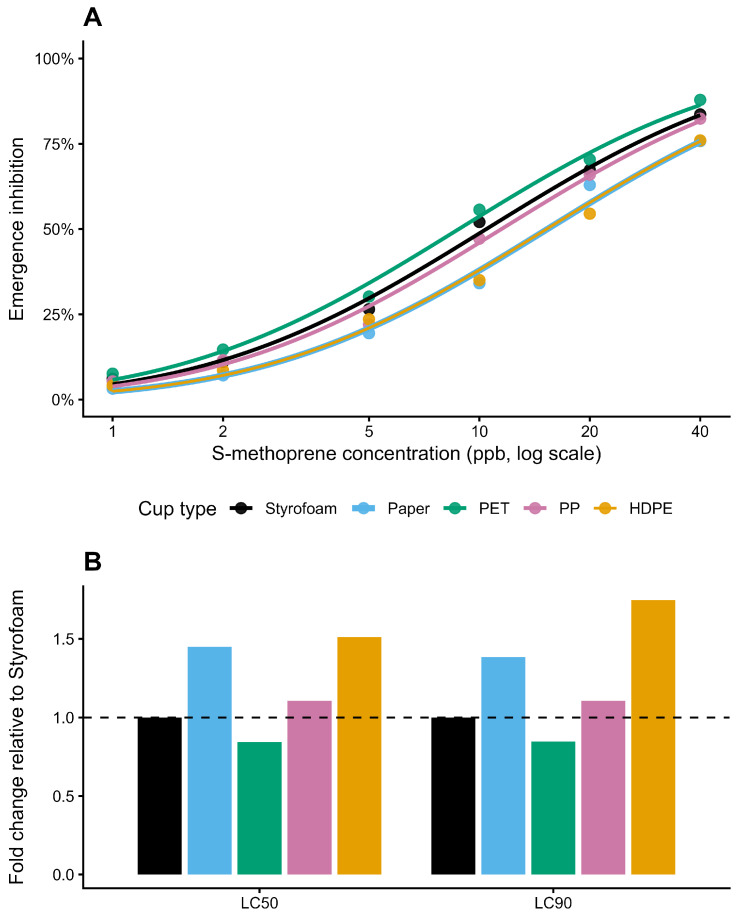
Effect of cup material on S-methoprene concentration-response relationships in *Culex quinquefasciatus* CQ1. (**A**) Probit concentration-response curves for mosquitoes exposed to S-methoprene in Styrofoam, polyethylene terephthalate (PET), polypropylene (PP), high-density polyethylene (HDPE), and paper cups. Points represent observed emergence inhibition pooled across assay repetitions, and lines represent fitted predictions from the generalized linear model. (**B**) Relative LC_50_ and LC_90_ estimates for each cup material expressed as fold change compared with the Styrofoam reference. Values greater than 1 indicate higher estimated concentrations required to achieve equivalent emergence inhibition relative to Styrofoam cups.

To compare each replacement cup material with the Styrofoam reference, planned contrasts were performed using estimated marginal means. For Figure 1, predicted concentration-response curves were generated from the main-effects model, and observed that emergence inhibition values were pooled across experimental replicates for each cup material and concentration. Figures were produced using the ggplot2 package [19]. The raw bioassay data are provided as Supplemental Dataset S1, and the R code used for the statistical analyses and figure generation is provided as Supplemental File S2.

## 3. Results

Control emergence inhibition was low across all cup materials, ranging from 0.6% in Styrofoam cups to 2.1% in HDPE, paper, and PET cups, with an overall control mortality of 1.5% (37/2472). S-methoprene concentrations ranged from 1 to 40 ppb and produced emergence inhibition ranging from 1.0% to 97.9%, encompassing nearly the full concentration-response curve (Figure 1A).

Cup material significantly affected the concentration required to achieve a given level of emergence inhibition (F(4, 140) = 12.34, *p* < 0.001). Relative to Styrofoam cups, HDPE (β = −0.274, *p* = 0.0001) and paper cups (β = −0.278, *p* < 0.0001) required higher concentrations of S-methoprene to achieve equivalent emergence inhibition, whereas PET (β = 0.125, *p* = 0.073) and PP cups (β = −0.069, *p* = 0.326) did not differ significantly from Styrofoam.

Estimated probit concentration-response slopes were similar among cup materials, ranging from 1.52 for HDPE cups to 1.69 for paper cups. Including a cup material × concentration interaction did not improve model fit (F(4, 136) = 0.388, *p* = 0.817), indicating no evidence that concentration-response slopes differed among cup materials.

Estimated LC_50_ values ranged from 8.3 ppb for PET cups to 13.8 ppb for HDPE cups (Table 1). Relative to the Styrofoam reference (LC_50_ = 9.4 ppb), LC_50_ estimates were 46% higher for HDPE cups, 40% higher for paper cups, and 18% higher for PP cups, and 12% lower for PET cups. Similar patterns were observed for LC90 estimates, which ranged from 48.3 ppb for PET cups to 75.5 ppb for HDPE cups. These differences are illustrated in Figure 1B. Consistent with the generalized linear model, HDPE and paper cups produced the largest increases in estimated LC values, whereas PET and PP cups produced LC estimates closer to those obtained using Styrofoam.

## 4. Discussion

Although PET produced results most similar to the Styrofoam reference, the broader finding is that assay container material can influence estimated LC values and therefore should be validated before routine use in resistance monitoring.

Concentration-response slopes were similar among cup materials and did not differ significantly, indicating that cup material primarily shifted the concentration required to achieve a given level of emergence inhibition rather than altering the overall concentration-response relationship.

One possible explanation is that differences in polymer composition, surface properties, or adsorption characteristics among cup materials altered the amount of S-methoprene available in the assay solution. S-methoprene is a hydrophobic juvenile hormone analog that readily adsorbs to certain substrates [12]. Although S-methoprene concentrations were not measured directly in this study, the consistent shift in LC values without corresponding changes in concentration-response slopes is consistent with reduced S-methoprene availability in some cup materials.

The findings of this study have important implications for mosquito control agencies conducting routine larval resistance monitoring. Larval susceptibility is commonly evaluated using resistance ratios, in which LC values for field populations are compared with those of a susceptible reference strain tested under the same assay conditions. In the present study, cup material shifted the concentration required to achieve a given level of emergence inhibition without altering the concentration-response slope. If a similar proportional shift appears to occur in field populations, cup material may have less influence on resistance ratios than on absolute LC estimates; however, this possibility was not evaluated. Consequently, replacement assay materials should be validated against established reference methods before implementation. When changes in assay materials are unavoidable, new baseline susceptibility values should be established for reference populations to maintain consistency in resistance monitoring.

Among the evaluated replacement materials, PET produced LC estimates closest to the Styrofoam reference and appears to be the most suitable replacement for S-methoprene larval bioassays. In contrast, HDPE and paper cups consistently produced higher LC estimates and may not maintain continuity with historical bioassay data. Polypropylene cups produced intermediate results; although they did not differ significantly from Styrofoam, LC estimates remained consistently higher than the Styrofoam reference. The differing results among PET, PP, and HDPE cups suggest that multiple material properties may influence S-methoprene availability within the assay system.

This study evaluated a single susceptible mosquito strain, one S-methoprene formulation, and laboratory bioassays conducted in distilled water. Consequently, the magnitude of the observed effects may differ among mosquito populations, formulations, or water chemistries encountered during routine resistance monitoring. In addition, S-methoprene bioavailability was inferred from biological responses rather than direct chemical measurements, and the proposed mechanism therefore remains hypothetical. Future studies should include direct chemical measurements of S-methoprene concentrations and evaluate additional mosquito populations, formulations, and water chemistries.

Despite these limitations, this study highlights the importance of validating replacement assay materials before they are adopted for standardized mosquito resistance bioassays. As regulatory and commercial changes continue to affect the availability of existing assay materials, alternative containers should be evaluated to ensure they do not introduce methodological bias into larval resistance monitoring.

## 5. Conclusions

HDPE and paper cups significantly increased estimated S-methoprene LC values, whereas PET produced results most comparable to Styrofoam. Because cup material can influence estimated LC values, replacement assay containers should be validated before adoption in mosquito resistance monitoring programs.

## Figures and Tables

**Table 1:** Estimated LC_50_ and LC_90_ values (ppb) and corresponding 95% confidence intervals for S-methoprene in laboratory larval bioassays conducted using five cup materials. Relative LC ratios are expressed relative to the Styrofoam reference (Styrofoam = 1.00). Values greater than 1 indicate that higher S-methoprene concentrations were required to achieve equivalent emergence inhibition than in Styrofoam cups.

Cup Material	LC_50_ (ppb)	95% CI	Relative LC Ratio	LC_90_ (ppb)	95% CI	Relative LC Ratio
Styrofoam	9.4	7.1–12.8	1.00	51.7	32.7–106.0	1.00
HDPE	13.8	10.9–18.0	1.46	75.5	49.4–142.0	1.45
Paper	13.2	10.3–17.7	1.40	68.8	44.2–136.0	1.33
PET	8.3	6.4–10.9	0.88	48.3	31.9–89.6	0.93
PP	11.1	8.4–15.2	1.18	59.9	37.7–125.0	1.16

## Data Availability

All data generated and analyzed during this study are provided in Supplemental Dataset S1. The R code used to reproduce all statistical analyses and figures is provided in Supplemental File S2.

## Supplementary Materials

The following supporting information can be downloaded at: https://www.mdpi.com/article/10.3390/pathogens15070769/s1, Supplemental Dataset S1: Raw emergence inhibition data from S-methoprene larval bioassays conducted using Styrofoam, polyethylene terephthalate (PET), polypropylene (PP), high-density polyethylene (HDPE), and paper cups; Supplemental File S2: R code used to generate LC50 and LC90 estimates, statistical analyses, and Figure 1 from Supplemental Dataset S1.

## Abbreviations

The following abbreviations are used in this manuscript:

CQ1	*Culex quinquefasciatus* reference colony
CI	Confidence interval
GLM	Generalized linear model
HDPE	High-density polyethylene
IGR	Insect growth regulator
IMM	Integrated mosquito management
JHA	Juvenile hormone analog
LC_50_	Concentration causing 50% emergence inhibition
LC_90_	Concentration causing 90% emergence inhibition
PET	Polyethylene terephthalate
PP	Polypropylene
WNV	West Nile virus
